# Pediatric Blastic Plasmacytoid Dendritic Cell Neoplasm: A Case Report

**DOI:** 10.1177/2632010X241304564

**Published:** 2024-12-16

**Authors:** Jasper X Zheng, Elham Vali Betts, Denis M Dwyre, Jong H Chung, Ananya Datta Mitra

**Affiliations:** 1 Department of Pathology and Laboratory Medicine, University of California Davis, Sacramento, CA, USA; 2 Department of Pediatrics, Division of Pediatric Hematology/Oncology, University of California Davis, Sacramento, CA, USA

**Keywords:** Blastic plasmacytoid dendritic cell neoplasm, plasmacytoid dendritic cells, Hispanic, mononuclear cells

## Abstract

Blastic plasmacytoid dendritic cell neoplasm (BPDCN) is a rare and aggressive neoplastic process of precursor plasmacytoid dendritic cells. The diagnostic evaluation of this heterogenous entity is challenging, requiring a comprehensive approach of incorporating clinical, morphologic, immunohistochemical, and molecular/cytogenetic evaluations. Optimal management of BPDCN remains controversial, and clinical outcomes continues to be poor. Pediatric cases of BPDCN are rare and to our knowledge, this is the second case of BPDCN described in a Hispanic child, first one was described outside the US in Peru. Here, we report a case of a juvenile patient of Hispanic origin presenting with cutaneous and bone marrow involvement and initially misdiagnosed as a cutaneous infection that resulted in subsequent delaying of necessary chemotherapy for 2 months. Biopsy of the lesion showed diffuse infiltration of immature cells involving the dermis with classical sparring of epidermis. A huge panel of immunohistochemical stains were performed to reach the diagnosis of BPDCN. Staging bone marrow biopsy also revealed involvement by BPDCN. Treatment was not only delayed in this patient but also due to the rarity of BPDCN in pediatric population, the subsequent therapeutic decisions were challenging for the primary oncology team as it was based solely on published literature on adult population. Our case report will not only add one more case in the pediatric age group, but also will also emphasize that although BPDCN has a grave prognosis in the elderly, timely diagnosis with prompt treatment is the key to complete remission in pediatric BPDCN population.

## Background

Blastic plasmacytoid dendritic cell neoplasm (BPDCN) is an exceptionally rare and aggressive neoplasm derived from abnormal clonal proliferations of precursor plasmacytoid dendritic cells.^1-3^ The precise pathogenesis of BPDCN is still not fully understood, it is believed to be associated with aberrant NF-kB activation.^4,5^ Diagnosis relies on combined morphologic and immunohistochemical demonstration of plasmacytoid dendritic cell characteristics with exclusion of other lineage-defining markers.^6,7^ Given the aggressive nature of this neoplastic entity and the lack of well-defined treatment options, early tissue biopsy and prompt diagnosis is preferred for timely medical intervention. Herein, we present a case of pediatric lower extremity unilocular cutaneous BPDCN that was initially misdiagnosed as an infectious manifestation.

## Case Presentation

**Figure 1.**
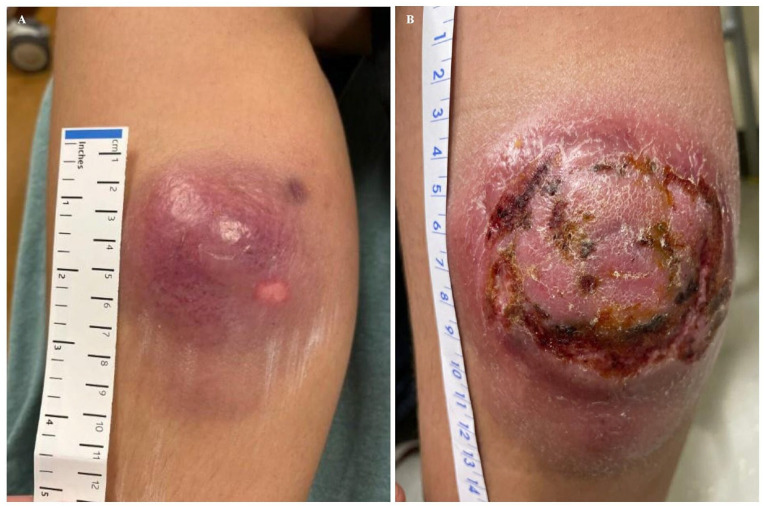
(A) Case at presentation at outside institution showing red non-tender induration and (B) showing rapid progression of the lesion within 3.5 months during presentation to our institution with 10 × 11 cm indurated red-purple nodule with central atypical erosion.

A 12-year-old Hispanic male with up to date on immunization and no significant past medical history developed a sizable progressively enlarging lesion on his right inner calf (Figure 1A) from an ecchymosis sustained from a ground level fall. Initial examination at an outside institution diagnosed the cutaneous violaceous lesion to be of infectious etiology. Incision and drainage was performed on the lesion and it drained clear fluid mixed with blood. Antibiotic (Cephalexin/Keflex, 500 mg) treatment was started, and reportedly it achieved initial improvement in appearance. Approximately a month after the initial encounter, the patient returned to the emergency department with a worsening lesional appearance. During this time, the patient did not have fever, pain, or restriction of movements. The patient was again treated with antibiotics and instructed to follow up with primary care physician. Three weeks later, the patient was referred to our institution for medical care. During this 3-week duration, the patient remained asymptomatic while maintaining his usual physical activities.

On examination, the right medial calf showed a persistent, large (11 cm × 12 cm) indurated red-purple cutaneous lesion (Figure 1B) with central erosion and focal areas of serosanguinous drainage. The extremities, however, appeared warm and well-perfused, without signs of edema. Night sweat was the only symptom endorsed. Otherwise, the patient appeared well, and was negative for gait disturbance, lymphadenopathy, hepatosplenomegaly, rash, or icterus. Laboratory findings (cell count with differential and basic metabolic panel) were all within the normal range. Punch biopsy of the skin lesion was performed soon after.

**Figure 2.**
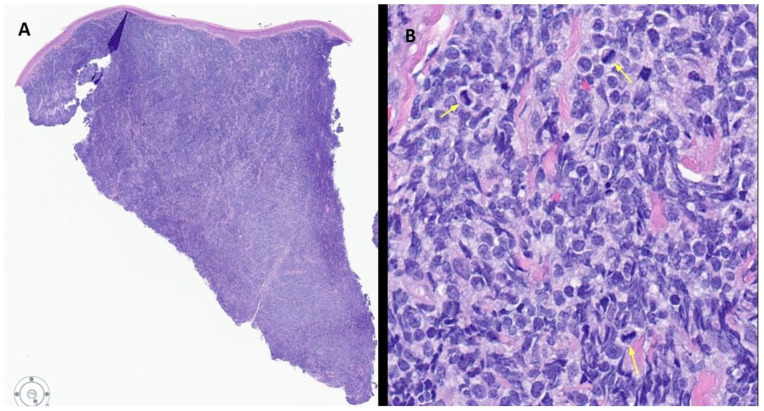
Skin punch biopsy of the right calf cutaneous lesion. (A) Low power view showing a diffuse atypical infiltrate sparing the epidermis and involving the dermis to the deepest aspect of the biopsy (H&E, ×20). (B) High power view showing the cells in the infiltrate are small to medium size with irregular nuclei, vesicular chromatin, inconspicuous nucleoli, and scant cytoplasm. Mitotic figures are frequently observed (yellow arrows indicating the mitotic figures). No necrosis noted. (H&E, ×400).

**Figure 3.**
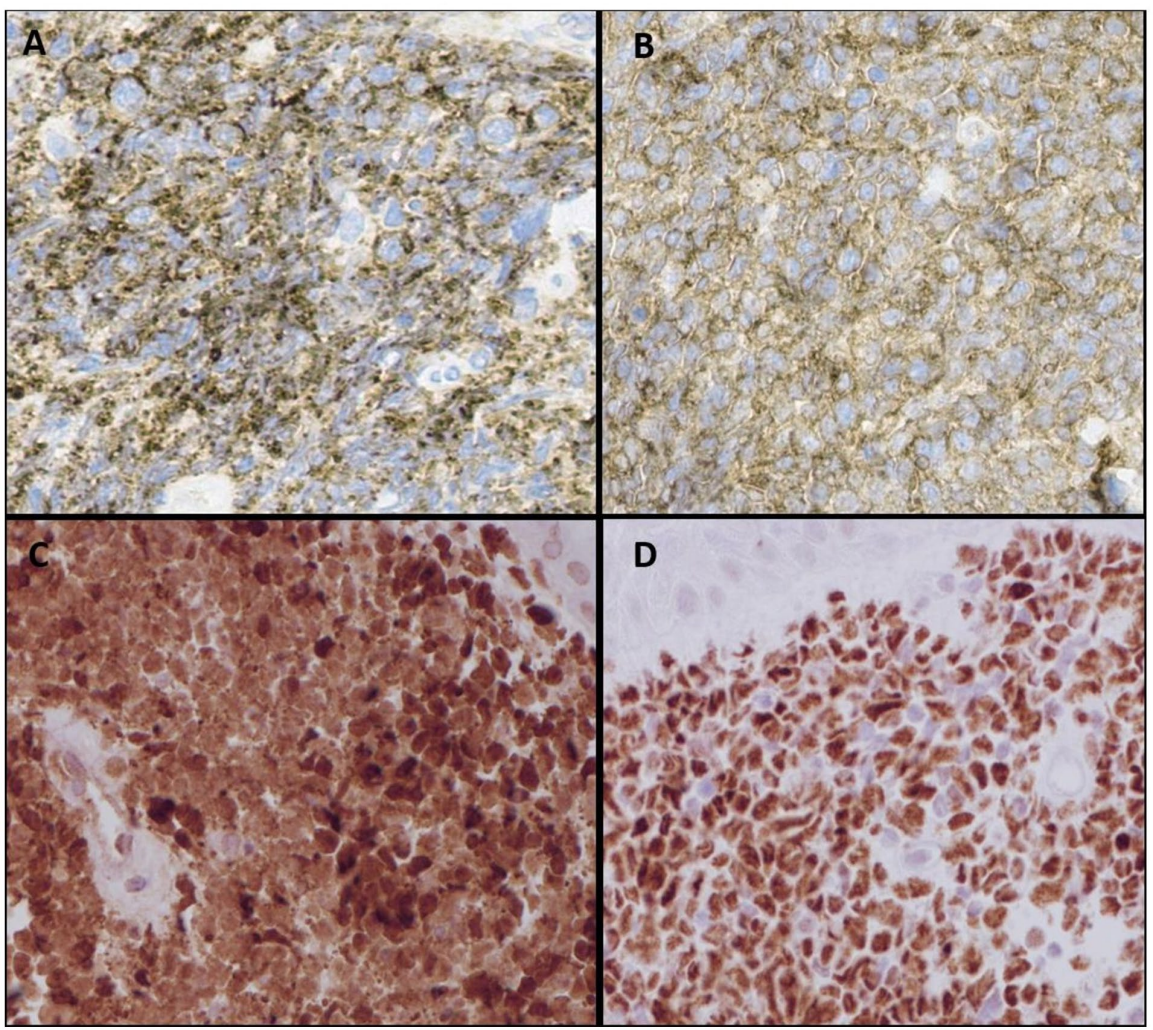
Skin punch biopsy (×400): (A) immunohistochemical staining for CD56 showing diffuse positivity, (B) immunohistochemical staining for CD123 showing diffuse positivity, (C) immunohistochemical staining for TCL-1 showing diffuse positivity, and (D) immunohistochemical staining for TCF4 showing diffuse positivity.

Histological examination of the skin punch biopsy specimen demonstrated diffuse infiltration of abnormal medium sized mononuclear cells that extended deep into the dermis with characteristic sparing of the epidermis (Figure 2A). These abnormal cells imparted an immature appearance with irregular nuclei, vesicular chromatin, inconspicuous nucleoli, and scant cytoplasm. Mitotic figures and apoptotic bodies were frequently observed; however, necrosis was absent (Figure 2B). Immunophenotypically, the abnormal cells were positive for CD4, CD56, CD123, TCL1, TCF4 (E2-2), CD43, BCL-2, and TDT. Partial CD99 and focal CD45 expression were also noted (Figure 3A–D). Specific markers of B cells, T cells, myeloid cells (CD33, CD34, CD117, MPO), and monocytic cells (lysozyme) were negative. The Ki-67 proliferation index was high (70%-80%). Together, the combined morphological features and immunohistochemical staining profile was consistent with the diagnosis of BPDCN.

**Figure 4.**
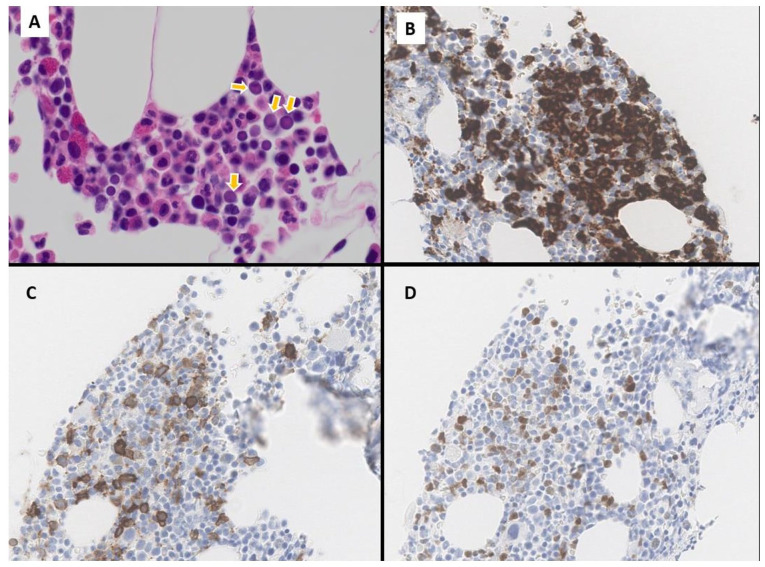
(A) Bone marrow trephine biopsy at diagnosis (H&E, ×1000), abnormal blastoid cells noted (yellow arrows) with immature morphology of high nuclear-cytoplasmic ratio, vesicular chromatin, and scant cytoplasm. (B) Bone marrow trephine biopsy at diagnosis (×400), immunohistochemical staining for CD123 reveals focal clustering of CD123+ cells. (C) Bone marrow trephine biopsy at diagnosis (×400), immunohistochemical staining for CD56 reveals focal clustering of CD56+ cells corresponding to abnormal cells seen on CD123 immunostaining. (D) Bone marrow trephine biopsy at diagnosis (×400), immunohistochemical staining for TCL1a.

**Figure 5.**
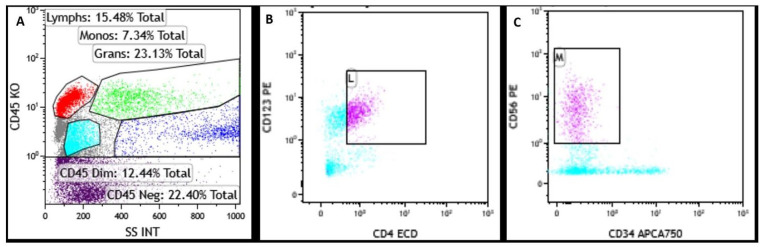
Flow cytometric analysis of a bone marrow aspirate sample at diagnosis. The abnormal blast population is positive for (A) CD45(dim), positive for (B) CD4+, CD123+, positive for (C) CD56++ and negative for CD34.

The subsequent staging with bone marrow trephine biopsy showed focally scattered metastatic tumor cells, highlighted by immunohistochemical staining for CD123, CD56, and TCL1a (Figure 4A–D) which approximated to 10-15% of marrow involvement by BPDCN (Figure 5A–C). Comprehensive myeloid next generation sequencing studies in the bone marrow did not show any obvious abnormalities. Examination of the cerebral spinal fluid demonstrated no discernible evidence of CNS involvement.

**Figure 6.**
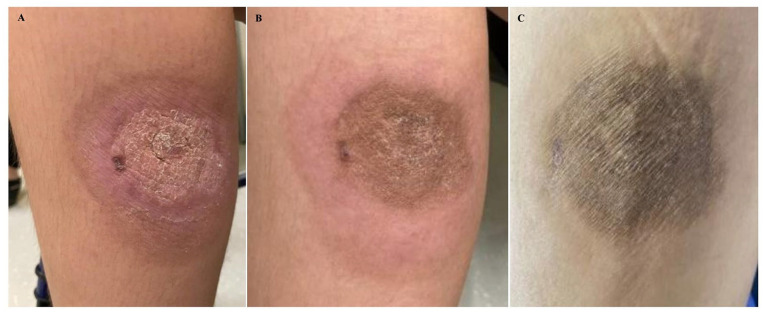
Photograph showing postinduction regression of right calf cutaneous lesion: (A) Day 27 of induction therapy, (B) Day 8 of consolidation after completion of initial induction with alternating cycles of Tagraxofusp infusion, and (C) Day 22 of consolidation.

Our patient responded well to the initial ALL-like regime induction chemotherapy (AALL1732 (HR) protocol) with alternating cycles of Tagraxofusp. The post-treatment cutaneous lesion demonstrated marked reduction in size, protuberance, and erythema (Figure 6A–C). Currently, the patient is disease free and is receiving maintenance therapy with mercaptopurine, vincristine, and methotrexate with intrathecal chemotherapy with cytarabine/hydrocortisone

## Discussion

BPDCN is a disease that is most frequently seen in adults with occasional documented cases in infants and children.^6,8^ The majority of pediatric patients present initially with cutaneous manifestations and only a minority with non-cutaneous manifestations (21%-24%).^2^ The relatively asymptomatic dermatopathic phase may involve any cutaneous body site with single or multiple variably sized red to bluish nodules or plaques ranging from a few millimeters to several centimeters.^6,8^ Hematologic dissemination is characterized by involvement of the bone marrow, peripheral blood, and/or lymph node(s).^6,7,9^ Patients may present with leukocytosis, anemia, thrombocytopenia, and circulating blasts mirroring acute leukemic manifestations. Less common disease manifestations include hepatomegaly, splenomegaly, and mucosal lesions.^6,8^ CNS dissemination without neurological signs is frequently discovered at diagnosis.^10^ Interestingly, the relapse rate does not vary between patients with and those without CNS involvement at initial diagnosis.^8,10^

Histologically, cutaneous involvement is characterized by dense monotypic neoplastic blastoid cell infiltration of the dermis with sparing of the epidermis and adnexal structures. Our patient’s skin biopsy studies were very typical showing dense dermal involvement with sparring of the epidermis. Lymph node infiltration commonly occurs in the interfollicular and medullary areas.^8,9^ Bone marrow involvement can range from focal interstitial infiltrations, tumor cell clusters, to confluent involvement with pronounced residual dysplastic megakaryocytes.^1,9^ The bone marrow involvement in the present case is also very focal and the cells were highlighted only by immunostains, no dysplastic megakaryocytes are noted.

Cytomorphologically, BPDCN is characterized as medium sized cells with a high nuclear-to-cytoplasmic ratio, scant basophilic cytoplasm, occasional small vacuoles, and pseudopodia resembling lymphoblasts, monoblasts, or myeloblasts. Nuclei are often single, with fine delicate chromatin and variably irregular nuclear contours. The proliferation rate is typically high, with readily identifiable active mitosis and apoptotic bodies.

Immunophenotypically, the blastoid neoplastic cells usually has co-expression of CD4 and CD56 with absence of certain B cell, T cell, myeloid, monocytic, and NK cell lineage markers.^11^ Rare cases may lack either CD4 or CD56, but never both.^6^ Plasmacytoid dendritic cell lineage should be demonstrated by expression of at least 2 of the following with certain degree of variability: CD123, TCF4, TCL1, CD303, CD2AP, BCL11a, and/or SPIB.^6,9,12^ Confident diagnosis of BPDCN requires a minimum of 4 of the following antigens: CD4, CD56, CD123, TCL1, TCF4, and CD303.^6,9,10,13-15^ For further confirmation, lineage markers of B cell, T cell, myeloid, monocytic, and NK cell (CD19, CD20, CD3, MPO, CD13, LAT, lysozyme, and CD16) should be excluded.^8^ The immunophenotypic pattern in our case was very typical of BPDCN and we ruled out B and T cell lymphomas and myeloid sarcomas and confirmed our morphologic diagnosis. In summary, if we see a blast like infiltrate in the dermal lesion biopsy which does not stain with the usual blast markers (CD34/CD117) and either B or T cell markers, we should definitely stain with CD4, CD56, CD123, TCL1 to reach this challenging diagnosis.

In flow cytometry studies, BPDCN neoplastic cells reside in the low-side scatter dim CD45 blast gate with a pathognomonic phenotype that must satisfy at least 2 of the following 4 criteria: (1) High-intensity CD123 expression, (2) BDCA2/CD303 expression, (3) BDCA4/CD304 expression, (4) CD4 positivity without MPO, cCD3, cCD79a, or CD11c expression.^6,11,13^ The bone marrow flow cytometry in our case showed high intensity CD123 expression with CD56 and CD4 positivity.

BPDCN has no defining pathognomonic or diagnostic cytogenetic alteration. The mutational abnormalities implicated in the pathogenesis of this entity affect a spectrum of functional classes of genes that includes DNA methylation, histone modification, signal transduction, transcription factors, cell-cycle regulation, and splicing factors.^5,16-19^ The most frequently mutated genes pertain to DNA methylation (*TET2* and, *IDH2*), chromatin remodeling (*ASXL1*), and RNA splicing (*ZRSR2* and, *SRSF2*).^20-22^ Other detectable mutations in BPDCN include: *TP53, IKAROS* family, *ETV6, NOTCH1, DNMT3A, KDM6A, SH2B3, ETNK1, HNRNPK, RAD21*, and the *RAS* pathway genes.^14,17,20,21^ The punch biopsy from this case was not submitted for any next generation sequencing studies, however, the next generation sequencing studies from the bone marrow aspirate did not show any pathogenic mutations, which could be due the focal nature of bone marrow involvement.

Suzuki et al^23^ discovered recurrent *MYB* rearrangement in both pediatric and adult BPDCN patients, with a frequency of 100% and 44%, respectively, however this was based on a very low number of patient population. Followed by most recent studies by Sakamoto et al^12^ showing a prevalence of MYC alteration (gene rearrangement and overexpression) in BPDCN approximately 40%. Given the rarity of the disease, determination of *MYB* rearrangements in pediatric BPDCN will not only expand diagnostic versatility but also provide a feasible conduit for patients to benefit from available molecular-targeting therapies for solid tumors with similar founder mutation/rearrangement.

The clinical course for BPDCN is typically aggressive, with a median overall survival of 9 to 20 months. Pediatric patients usually have more favorable survival rates and achievable complete remission lasting several years.^6,24^ Poor prognostic factors include older age of presentation, presence of at least 3 genetic mutations, abnormal transcription factors (TP53/RAS.81), and mutated DNA methylation genes (*TET2, IDH2*).^6,8,25,26^ *TET2* truncating mutations, biallelic deletion of *CDKN2A/CDKN2B* on 9p21.3, or mono-allelic loss of 5q31 are reported to be associated with worse survival outcomes.^16,20,21,27,28^ In contrast, *MYC* rearrangement, although only in a limited number of BPDCN patients, is associated with a positive response to acute lymphoblastic leukemia-type chemotherapy regimens.^12,29^

The optimal standardized treatment for BPDCN is currently not well-defined. Rigorous acute lymphoblastic leukemia/lymphoblastic lymphoma (ALL/LBL) induction therapy followed by allogeneic bone marrow transplantation (SCT) during first remission has demonstrated improved survival in pediatric population.^6,30^ However, due to the paucity of cases, the utility of allogeneic hematopoietic stem cell transplantation in first remission remains unclear for the pediatric patients.^18^ CNS, serving as a sanctuary for blasts, is likely the origin of relapse; regular prophylactic intrathecal chemotherapy is necessary to maintain remission in these patients.^31^ Innovative therapy targeting the ubiquitous aberrantly overexpressed interleukin-3 receptor alpha/CD123 with the immunotoxin SL-401 and the more recent anti-CD123 CAR-T therapy have reported promising results.^32-34^ Finally, exploration of the abnormally activated NF-kB pathway as a target for developing therapies is currently ongoing.^5,35^

Our patient did extremely well with his induction chemotherapy regimen and is in complete remission almost 2 years after his initial diagnosis and is yet to complete 2 more cycles of his maintenance therapy. To our knowledge, this is one of the few cases of BPDCN described in a Hispanic child, with the first one described outside the USA in Peru.^26^

## Conclusion

BPDCN is rare in pediatric age group and is extremely challenging to diagnose even with an extensive histopathologic examination. Given the challenging nature of this entity, appropriate and timely treatment can be delayed owing to the initial deceptively mild presentation which is further compounded by the lack of clear pediatric-focused therapeutic recommendations. It is worth emphasizing the importance of timely diagnosis with biopsy and prompt subsequent management as the first step toward a promising outcome.

**Pre-print version:** A preprint has previously been published [Jasper X Zheng et al, May 2023].^36^
